# A successful model for KSA-based payor-provider collaborative in maternity care

**DOI:** 10.1186/s41687-026-01142-w

**Published:** 2026-07-03

**Authors:** Abdulrehman Taher, Serena Sarkis, Hussein H. Khachfe, Fares Saade, Jason Greich, Eman ElBadrawy, Dalal AlHamed

**Affiliations:** 1 Value Based Care & Health Center, Bupa Arabia and Bupa Care Connect, Jeddah, Saudi Arabia; 2 Division of Value Based Healthcare, Capadev LLP, London, UK

**Keywords:** Maternity, VBHC, Implementation, Payor-Provider

## Abstract

**Background:**

Maternity care in the Kingdom of Saudi Arabia (KSA) faces rising Cesarean sections (C-section) rates, fragmented care pathways, and a lack of standardized outcome measurement. This study evaluates the feasibility and early outcomes of a large-scale payer–provider Value-Based Healthcare (VBHC) pilot integrating Patient-Reported and Clinician-Reported Outcome Measures (PROMs and CROMs) across four tertiary private hospitals with active maternity/obstetrics departments in KSA.

**Methodology:**

A multicenter outcomes rollout pilot was conducted across four tertiary hospitals with dedicated maternity/obstetrics departments in KSA between July 2024 and July 2025. The International Consortium for Health Outcomes Measurement (ICHOM) Pregnancy and Childbirth Standard Set PROMs and CROMs were digitally collected at five predefined control points using a centralized interoperable platform from pregnant women aged 18–45 years. Site Rollout Teams were trained in VBHC principles, patient journey mapping, and outcomes utilization. Response rates, longitudinal PROMs trends, minimum important difference (MID) analyses, and PROM–PROM, PROM–CROM, and CROM–CROM correlations were evaluated.

**Results:**

A total of 802 women were enrolled, with an overall PROMs response rate of 38.7%. While mean PROMs changes remained below MID thresholds at the population level, approximately one-third of women achieved clinically meaningful individual improvements across key domains, including mental health, continence, breastfeeding confidence, and bonding. Hospitals implementing PROMs-triggered referral pathways demonstrated substantial reductions in abnormal outcome rates. Meaningful correlations were observed across psychosocial, functional, and clinical outcomes, supporting internal data validity and clinical coherence. Two providers achieved ICHOM Level 1 accreditation by project completion.

**Conclusions:**

This pilot demonstrates that standardized PROMs and CROMs integration into routine maternity care is feasible and operationally effective in Saudi Arabia. Payer–provider collaboration, digital infrastructure, and clinician engagement enable actionable, patient-centered insights and establish a scalable foundation for VBHC-aligned maternity care and payment reform.

**Supplementary Information:**

The online version contains supplementary material available at https://doi.org/10.1186/s41687-026-01142-w.

## Background

Saudi Arabia’s Vision 2030 places healthcare transformation at the forefront of national priorities, with the value-based healthcare (VBHC) model prioritized to achieve maximum quality and sustainability [1]. Despite heavy investments in infrastructure and digital health, the current healthcare delivery model remains largely volume-based, with fee-for-service reimbursement systems that reward activity rather than outcomes. This has been one of the main drivers behind escalating healthcare expenditure and variable quality across institutions. In maternity care, rising C-section rates, wide patient outcome variations, and fragmented care pathways point to the need for a more patient-centered and outcomes-based model of care[2–4].

Globally, the implementation of VBHC frameworks in maternity services has gained significant momentum over the past decade. ICHOM’s Pregnancy and Childbirth Standard Set, introduced in 2016, provided the first internationally standardized blueprint for capturing patient-reported and clinical outcomes across the full maternity pathway, and has since been adopted across health systems in Europe, North America, and parts of Asia [5]. Payer-driven VBHC initiatives, particularly those linking outcome measurement to provider contracting and bundled payment models, have demonstrated measurable improvements in maternal and neonatal outcomes in countries such as the Netherlands, Sweden, and the United States [6]. Despite this global trajectory, the Gulf Cooperation Council (GCC) region, and Saudi Arabia in particular, has lacked structured, large-scale payer–provider collaborations built around standardized maternity outcome measurement. Existing maternity care quality initiatives in KSA have largely focused on process indicators and mortality metrics, without systematic integration of Patient-Reported Outcome Measures (PROMs) or longitudinal outcome tracking across the care continuum. This represents a significant gap: Saudi Arabia’s ambitious Vision 2030 health transformation agenda and the Council for Health Insurance’s National PROMs strategy both call for outcomes-driven care, yet no scalable model for achieving this in maternity had been tested prior to this initiative [7]. Accordingly, Bupa Arabia, the largest private health insurer in the GCC region took the leading position in testing these VBHC principles in the Saudi market to fill this gap. This pilot, conducted in partnership with Capadev LLP, one of the International Consortium for Health Outcomes Measurement (ICHOM) approved implementation and accreditation partners, was designed to integrate international VBHC frameworks into the health system of Saudi Arabia. The initiative sought to: (1) align care pathways and outcome measures to enable effective benchmarking; (2) implement systematic digital collection of PROMs and Clinician-Reported Outcome Measures (CROMs) based on ICHOM standard sets; (3) convert outcome data into actionable information for decision-making, enhance quality of care, and tailor women’s needs during the journey of maternity care; (4) integrate standardized outcome measures as the evidential basis for issuing Bupa Arabia’s Centers of Excellence designation — a formal recognition awarded to provider institutions that meet defined quality and outcome benchmarks, and which carries implications for preferential network positioning and patient referral incentives within Bupa Arabia’s insured population; and (5) explore opportunities for innovative value-based payment models, such as bundled payments, that incentivize quality and efficiency rather than volume.

The research hypothesis is that implementing VBHC principles is both feasible and effective in the Saudi context, enabling standardized, patient-centered, data-driven maternity care aligned with national VBHC ambitions.

## Methods

This study was conducted in Saudi Arabia between July 2024 and July 2025 as a multicenter, prospective outcomes measurement pilot. Four private tertiary hospitals with active maternity and obstetrics departments participated, located across three regions: Riyadh, Al Ahsa (including Dammam, Al Jubail, Al Khobar, and Muhavil), and Jeddah. Participating hospitals were selected by Bupa Arabia — the largest private health insurer in Saudi Arabia — based on patient volume, clinical leadership commitment, institutional readiness for digital health integration, and existing quality improvement infrastructure. The initiative was conceived and strategically driven by Bupa Arabia, serving as the lead governing body and primary sponsor. Bupa Arabia orchestrated the project’s design and funding, partnering with Capadev LLP—an ICHOM-approved implementation partner—to execute the technical and clinical deployment. The pilot was designed as an operational feasibility and outcomes measurement study, with the primary objective of embedding standardized PROM and CROM collection into routine maternity care pathways at each participating site.

### Pre-launch planning

Prior to data collection, each participating hospital established a Site Rollout Team (SRT) comprising physicians, nurses, and quality representatives, tasked with leading the initiative at the institutional level. SRT members participated in a blended online and in-person training course on VBHC principles, equipping them with the foundational knowledge and practical competencies required to lead outcome collection and utilization at their respective sites. Upon successful completion of the course, members received a formal certificate. Each SRT subsequently developed a Patient Journey Map — a structured visual tool mapping the sequence of patient interactions and clinical touchpoints across the maternity pathway — drawing on global best practices to define the optimal timing and content of outcomes collection across the care continuum.

### Outcomes rollout preparation

To comprehensively track maternal health across the pregnancy journey, ten validated PROM instruments were selected from the ICHOM Pregnancy and Childbirth Standard Set, administered across five predefined Control Points (CPs) — standardized moments in the maternity pathway at which outcomes data were collected, corresponding to clinically meaningful care transitions. The instruments covered the following domains: general health-related quality-of-life (EQ-5D-3L), sexual functioning (PROMIS SexFS), antepartum and postpartum depression and anxiety screening (PHQ-2), urinary incontinence (ICIQ), stool incontinence (Wexner), birth satisfaction (BSS-R), mother-infant bonding (MIBS), breastfeeding self-efficacy (BSES), social support (SIMSS), and deep postpartum depression (EPDS), as illustrated in Fig. 1. The EQ-5D-3L was selected as the generic health-related quality of life measure in accordance with its specification in the ICHOM Pregnancy and Childbirth Standard Set. The 3L version was preferred over the 5L given its established use in the ICHOM Standard Set at the time of implementation. EQ-5D was selected over the PROMIS Global-10 because EuroQol supports fully localized, population-specific language validation — including a Saudi Arabian version — whereas PROMIS provides only a generalized Arabic translation without regional normative calibration, which would have reduced comparability within the KSA population context. All instruments were localized following International Society for Pharmacoeconomics and Outcomes Research (ISPOR) translation and cultural adaptation guidelines and validated for Saudi cultural relevance through Acolad, an ICHOM-approved translation partner. CROMs were selected in parallel based on the same ICHOM Standard Set, and case-mix variables were defined in accordance with the Standard Set’s specifications to ensure consistency with global benchmarking practice.

**Fig. 1:**
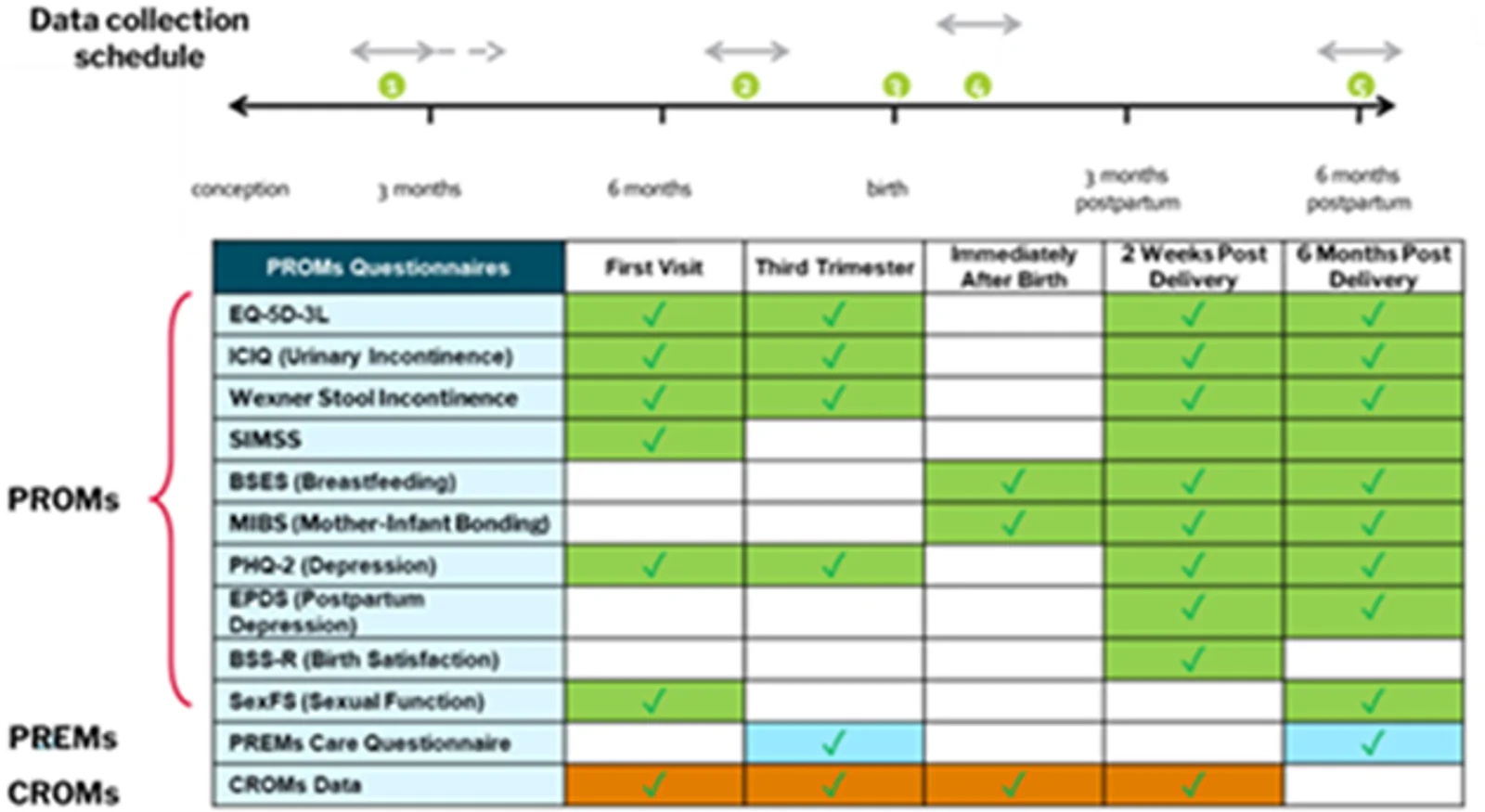
Outcomes to be collected and control point schedule

The five Control Points were structured as follows: CP1 — the point of registration into the care pathway, occurring at any time before 24 weeks of gestation; CP2 — the third trimester visit; CP3 — immediately post-delivery; CP4 — two weeks post-delivery; and CP5 — six months post-delivery.

Patient inclusion criteria were defined to ensure population comparability across providers and to support pilot standardization. Eligible women were Arabic-speaking, aged 18 to 45 years, and residents of Al Ahsa, Riyadh, or Jeddah. Women were required to be registered into the care pathway before 24 weeks of gestation. The age range of 18 to 45 years was applied to standardize the pilot cohort, and to ensure appropriate ethical and informed consent procedures were followed; the inclusion of all age groups is planned for subsequent scale-up phases. Exclusion criteria included pre-existing comorbidities (diabetes, sickle cell disease, cardiovascular conditions including heart failure and coronary artery disease, and mental illness), non-native Arabic speakers, and women registering at or beyond 24 weeks of gestation. Physicians treating enrolled women were required to be full-time staff at the participating institutions for the duration of the pilot; part-time physicians were excluded for the first two months of the project.

### Data collection solution launch

The ViHA (Value-in-Health Agent) platform — Capadev’s 360° interoperable outcomes management system — was configured and deployed on a Level C KSA-compliant local server in accordance with national data residency and security requirements. The platform was implemented across all four provider sites to enable fully digital, automated outcomes collection. PROM assessments were automatically dispatched to enrolled women via their preferred communication channel (SMS, WhatsApp, or email) at each scheduled Control Point. Each assessment incorporated an informed consent step and a data confidentiality assurance. The platform aggregated all incoming data in real time, enabling continuous analysis, dashboard reporting, and provider- and physician-level outcome monitoring.

### Outcomes rollout

A structured implementation plan was co-developed with clinical and operational stakeholders at each participating site prior to data collection going live. Stakeholders included OBGYN department chairs, chief medical officers, quality directors, nursing leads, and IT representatives at each hospital, alongside the Bupa Arabia and Capadev implementation teams. Co-development sessions were conducted in the form of structured workshops and steering committee meetings, through which site-specific patient communication protocols, staff responsibilities, and escalation pathways were agreed upon and documented.

The patient-facing communication plan defined the channel, timing, content, and language of all PROMs outreach across the five Control Points. Women were contacted via their preferred digital channel — SMS, WhatsApp, or email — at each scheduled assessment window, with up to two automated reminders issued for non-responses. Reminder cadences were calibrated per Control Point, with shorter follow-up intervals at postpartum stages where drop-off risk was highest. All patient-facing communications were available in Saudi Arabic and included a brief explanation of the purpose of the assessment and a direct link to the consent-gated digital questionnaire.

Clinical staff onboarding was structured through the SRT framework established during Pre-Launch Planning. Site-specific onboarding sessions reinforced staff roles in PROMs workflow integration — including how to prompt patients at appointments, manage flagged threshold alerts, and upload CROMs within the ViHA platform. Role-specific training materials and reference guides were distributed to all participating clinicians and administrative staff.

Data governance was maintained throughout the collection period by the Capadev clinical monitoring team, which conducted regular site-level data quality reviews, flagged incomplete or anomalous entries, and coordinated directly with SRTs to resolve discrepancies at source. Formal data quality checks assessed completeness, internal consistency, and timeliness of both PROMs and CROMs entries at each Control Point. Any site deviating materially from collection protocols was identified and supported through a structured remediation process.

### Dashboarding and ramp-up

Outcome dashboards were developed and deployed within the ViHA platform by Capadev as the platform vendor and primary implementation partner (Fig. 2, illustrative). Dashboard configurations were co-designed iteratively with hospital clinical and quality teams through a series of design review sessions, ensuring that the outputs were structured around clinically meaningful use cases rather than data display alone. Three distinct dashboard tiers were configured to serve different target audiences. Clinician-facing dashboards provided individual patient-level PROMs score tracking, automated threshold alerts triggering referral prompts, and longitudinal outcome trajectories across the five Control Points, enabling personalized, responsive care at the point of contact. Quality and department-level dashboards provided aggregated site-level outcome profiles, enabling department heads and quality leads to monitor population-level trends, identify high-risk patient subgroups, and track the impact of referral pathway activation over time. Hospital management and Bupa Arabia-facing dashboards provided cross-site benchmarking outputs, response rate performance, and program-level outcome summaries, supporting governance, accountability, and value-based contracting discussions. No patient-facing dashboard views were deployed in this pilot phase; direct patient access to outcomes data is planned for future iterations of the program.

**Fig. 2:**
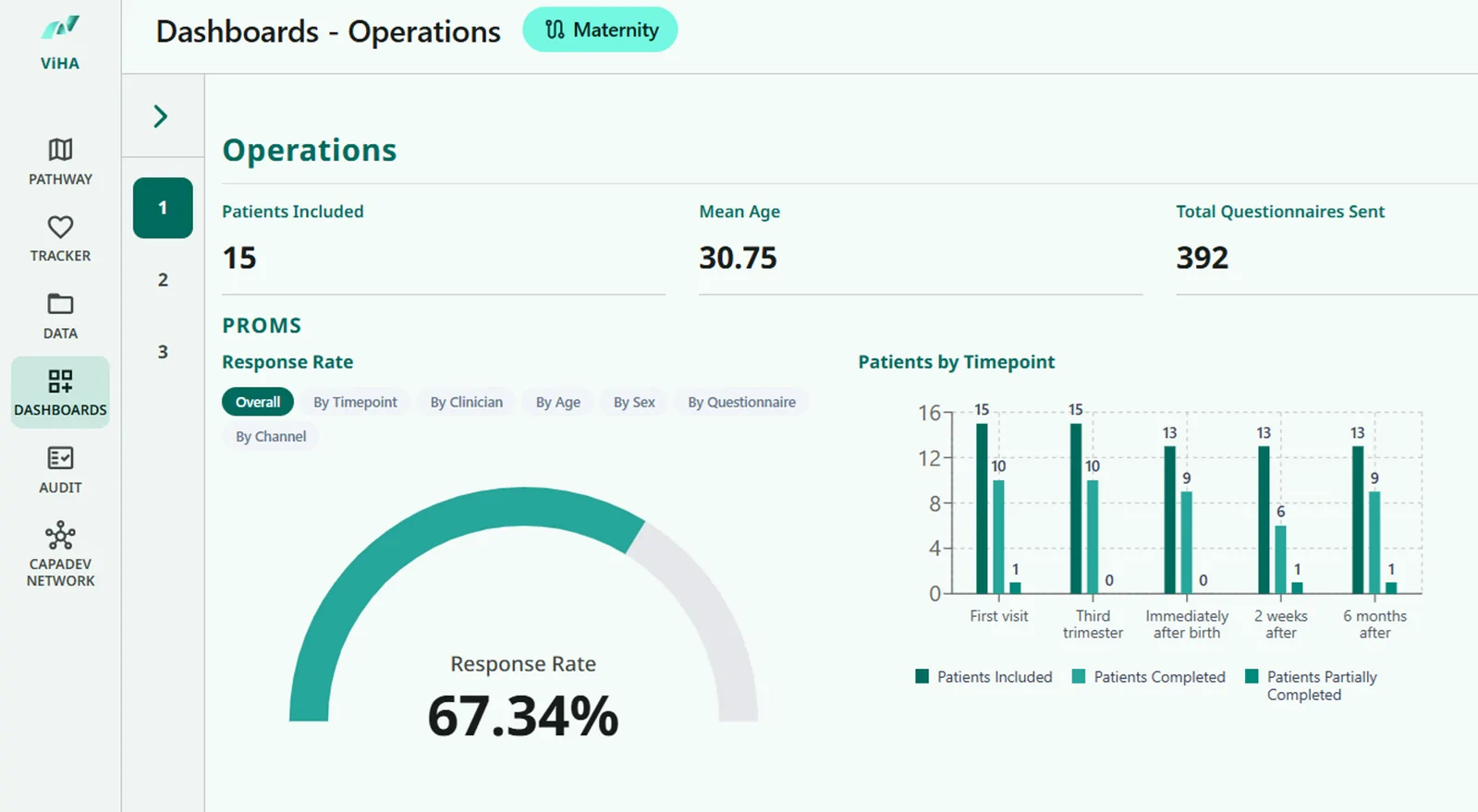
Digital platform dashboards (Illustrative)

### Accreditation

Throughout the pilot, data quality was continuously assessed for completeness, validity, and accuracy in accordance with ICHOM accreditation requirements. By the conclusion of the project period, two of the four participating hospitals had met the minimum criteria for ICHOM Level 1 Accreditation — a formal designation recognizing that an institution systematically collects, reports, and uses outcomes data in line with ICHOM standards. Capadev supported both institutions in preparing and submitting the required documentation, and both were successfully accredited. The remaining two hospitals did not meet the criteria for ICHOM Level 1 Accreditation by the conclusion of the pilot. In both cases, insufficient PROMs collection at the provider site was the primary barrier, driven by a combination of limited physician engagement, suboptimal patient activation, and inadequate adoption of the structured follow-up techniques and patient outreach strategies developed during the Pre-Launch Planning phase. These institutional gaps resulted in response rates and data completeness levels that fell below the minimum thresholds required for accreditation review. ICHOM accreditation was positioned by Bupa Arabia as a meaningful incentive for provider participation, reflecting the insurer’s intent to link Centers of Excellence designation to evidence of standardized, outcomes-driven care delivery.

### Data analysis

PROMs scores were calculated in accordance with the scoring manuals of each respective instrument. For the EQ-5D-3L, utility index scores were derived using the validated Saudi Arabic value set where available; in its absence, the closest culturally proximate published tariff was applied (Iran in this case). For all other instruments, published scoring algorithms were followed as specified by the instrument developers. CROM variables were extracted directly from the ViHA platform as uploaded by clinical staff and verified against source records during quality monitoring.

Response rates were calculated as the proportion of enrolled women who completed at least one item of a given PROMs at each Control Point, divided by the total number of women eligible for assessment at that point. Overall PROMs response rate was calculated as the mean completion rate across all instruments and all Control Points.

The clinical significance of observed PROMs score changes was evaluated using the Minimum Important Difference (MID) framework. MID thresholds were sourced from published literature for instruments with established benchmarks. For instruments without published MID values applicable to a maternity population, the threshold was estimated using the distribution-based method of 0.5 × the standard deviation of baseline scores, consistent with established methodological guidance [8]. A change in the direction of a better outcome that exceeded the MID threshold was classified as a clinically meaningful improvement; a change in the direction of a worse outcome exceeding the MID threshold was classified as a clinically meaningful decline. Both population-level mean changes and individual-level MID responder rates — defined as the proportion of women whose personal score change exceeded the MID threshold — were reported.

Correlations between PROMs instruments (PROM–PROM), between PROMs and CROMs (PROM–CROM), and between CROMs (CROM–CROM) were assessed using methods appropriate to variable type: Spearman’s Rank Correlation for continuous and ordinal variables, Phi Coefficient for binary versus binary comparisons, Point-Biserial Correlation for binary versus continuous or ordinal variables, and Cramér’s V for categorical versus categorical comparisons. Only associations meeting a threshold of |r| ≥ 0.20 with clear clinical relevance or alignment with established literature were reported, given the exploratory nature of the analysis and the limited sample size at this stage. All analyses were conducted using Python. Missing data were handled by complete-case analysis; no imputation was performed.

## Results

### Response rates

#### Per control point

Over the course of one year, a total of 802 pregnant women were onboarded, with 38.7% completing assessments and 44.3% having CROMs uploaded on their behalf (Fig. 3). Response rates decreased at later stages of the maternity journey.

**Fig. 3:**
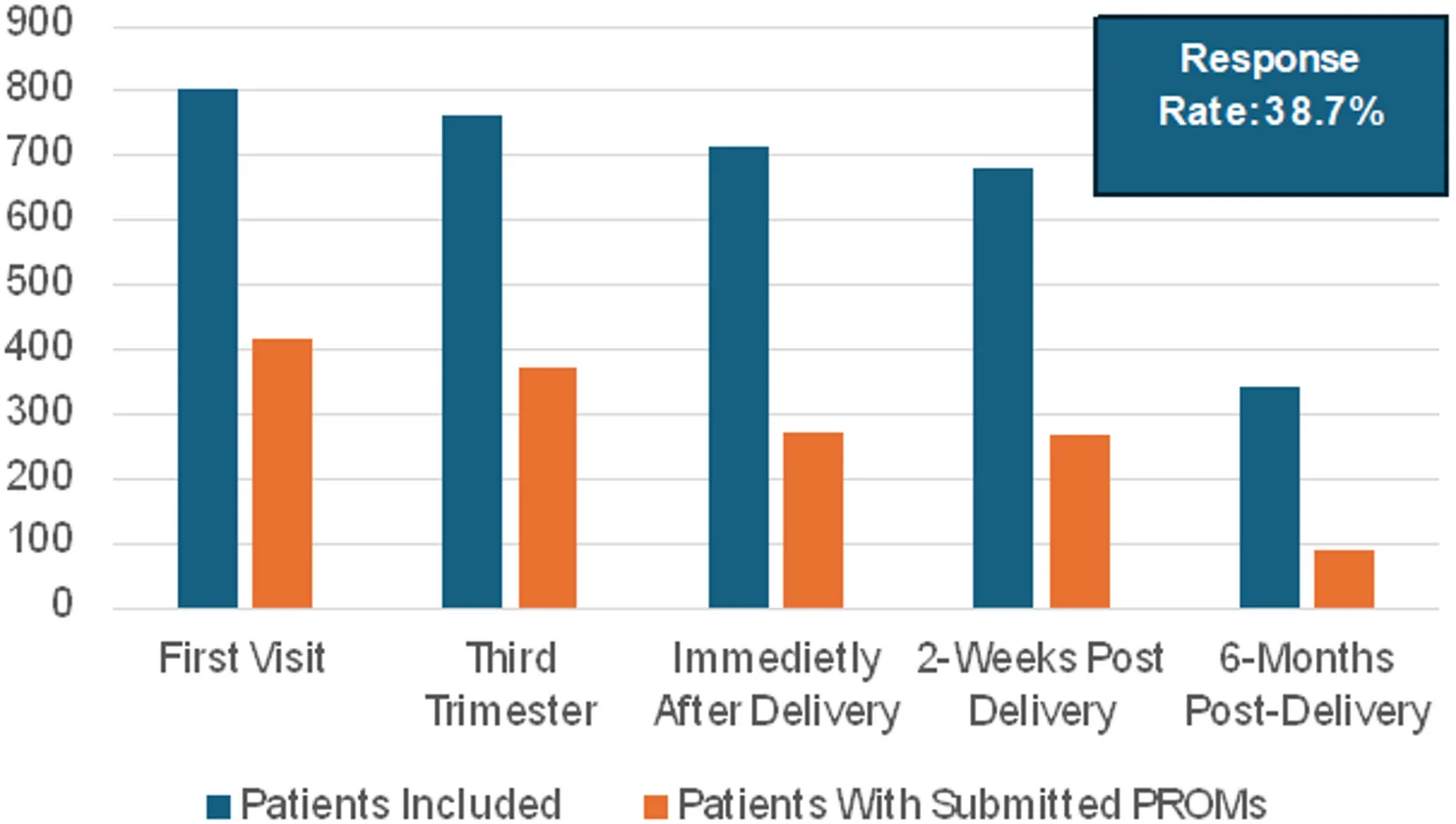
Average PROMs Response Rate per Control Point

#### Per questionnaire

Analysis of response rates across different PROMs categories revealed varying levels of participation (Fig. 4).

**Fig. 4:**
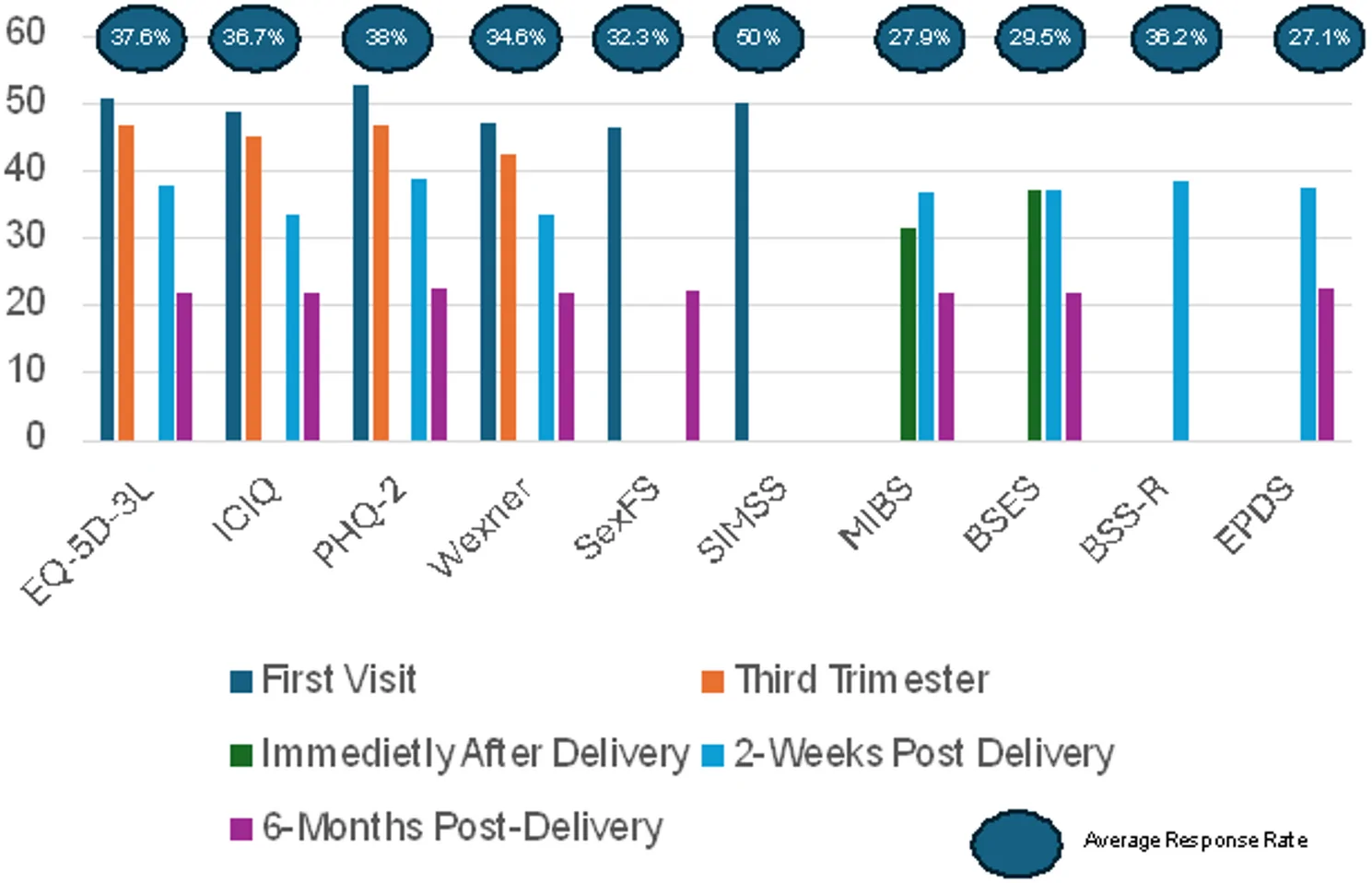
Average PROMs response rate per questionnaire

To illustrate, PROMs related to Depression and Sexual Functioning and Satisfaction have the highest response rate of around 50%.

In contrast, PROMs for post-partum depression and mother-infant bonding score recorded the lowest response rates, dropping to a low of 31% in some instances.

#### Per hospital

Overall response rates among providers in the pilot program have ranged from 29.5% to 58% as shown in Fig. 5.

**Fig. 5:**
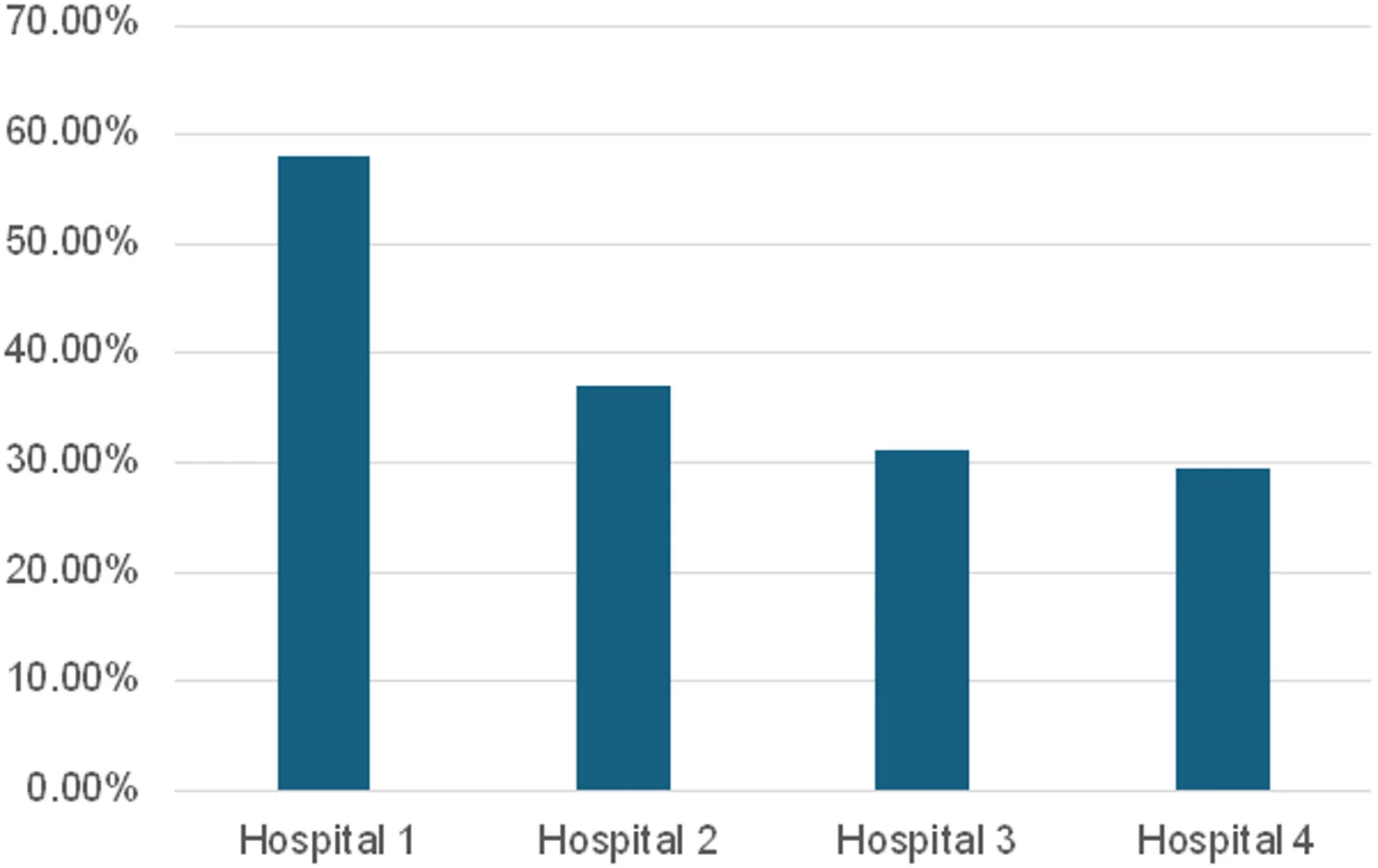
Overall response rate across different hospitals in pilot

### Scores and minimum important clinical difference (MID)

EQ-5D-3L scores remained stable during pregnancy and showed modest improvement post-partum (**0.77** → **0.77** → **0.81** → **0.83**). The change from CP2 to CP4 (**Δ = +0.04**) did not exceed the MID threshold of **0.085**, indicating no clinically meaningful group-level change. However, approximately **38%** of women experienced clinically significant improvement individually.

ICIQ scores increased slightly during pregnancy and improved after birth (**1.99** → **2.27** → **1.90** → **1.80**). The reduction from CP2 to CP4 (**Δ = − 0.37**) and CP2 was below the MID threshold of **1.5**, meaning no clinically meaningful change at the population level. Nonetheless, around **27%** of women experienced individually meaningful improvement.

PHQ-2 scores showed slight improvement antenatally and minor increases postpartum (**1.22** → **1.11** → **1.24** → **1.42**). The change from CP2 to CP4 (**Δ = +0.13**) was well below the MID threshold of **1.5**, indicating no clinically meaningful mean-level change. Approximately **25%** of women exceeded the MID individually.

WEXNER scores improved modestly across pregnancy and postpartum (**4.59** → **3.70** → **3.70** → **3.45**). The change from CP2 to CP4 (**Δ = 0.00**) did not exceed the MID threshold of **1.9**. While the overall change was not clinically meaningful, approximately **one-third** of women achieved improvements exceeding the MID individually.

SexFS scores remained largely stable across CP1 and CP5 (**2.07** → **1.80**). The change (**Δ = − 0.27**) was below the MID threshold of **0.53**, indicating no meaningful change at the group level. Around **25%** of patients achieved clinically significant individual improvement.

SIMSS was collected only at CP1, with an average first-visit score of **2.84**. No longitudinal comparisons were available.

MIBS scores improved slightly between delivery and two weeks postpartum before stabilizing (**3.04** → **2.79** → **3.00**). The CP3→CP5 change remained below the MID threshold of **1.9**, indicating no clinically meaningful mean-level improvement. However, **38%** of women demonstrated clinically meaningful individual improvement.

BSES scores were stable from delivery to two weeks postpartum but declined by six months (**45.41** → **45.81** → **39.79**). The CP3→CP5 change (**Δ = − 5.62**) remained below the MID threshold of **7–8 points**, indicating no meaningful population-level change. Nonetheless, **38%** of women exceeded the MID threshold individually.

BSS-R was collected only at CP4, with an average score of **26.52**, and thus no change analysis was possible.

EPDS scores remained virtually unchanged between CP4 and CP5 (**7.21** → **7.23**). The minimal change (**Δ = +0.02**) was far below the MID threshold of **4 points**, indicating no clinically meaningful change at the group level. Approximately **32%** of women achieved MID-level improvement individually.

### Correlations

Preliminary analysis of correlations between PROMs instruments identified several statistically meaningful associations, as shown in Fig. 6 and Supplementary Table 1:

**Fig. 6:**
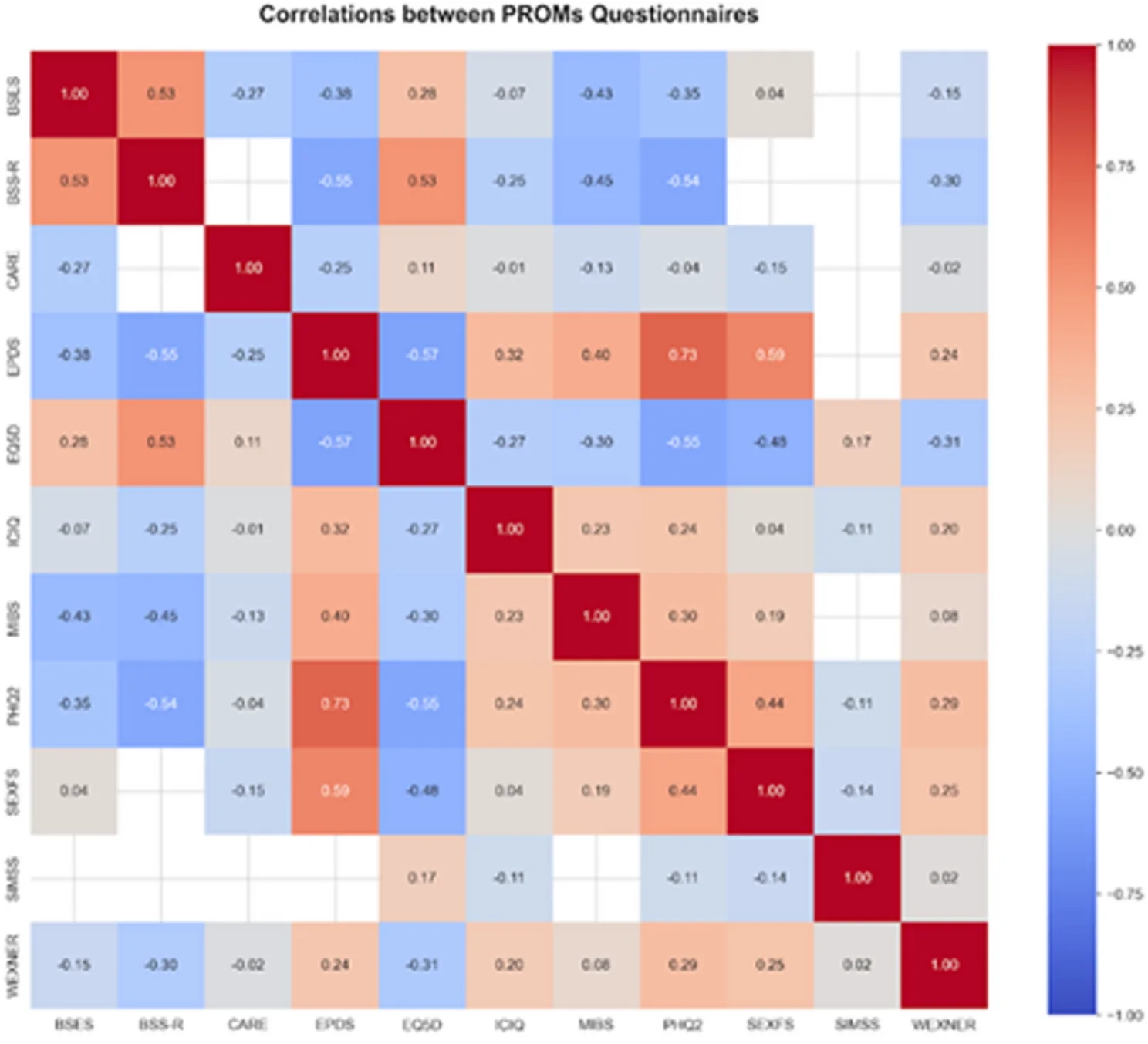
PROM-PROM Correlations

A number of correlations were observed:

- EPDS correlated with PHQ-2 and SexFS (both *r* = 0.59), and negatively with EQ-5D-3L (*r* = -0.57).
- EQ-5D-3L showed negative correlations with PHQ-2 (*r* = -0.55) and positively with SexFS (*r* = 0.48).
- BSS-R correlated positively with EQ-5D-3L (*r* = 0.53) and negatively with EPDS (*r* = -0.55) and PHQ-2 (*r* = -0.54).
- BSES correlated negatively with EPDS (*r* = -0.38) and PHQ-2 (*r* = -0.35).
- MIBS correlated negatively with BSES (*r* = -0.43) and BSS-R (*r* = -0.45), and positively with EPDS (*r* = 0.40).
- SexFS correlated positively with PHQ-2 (*r* = 0.44).

Among the preliminary correlations analyzed between PROMs and CROMs, several associations were identified as shown in Supplementary Table 2:

- Wexner correlated negatively with maternal length of stay (*r* = -0.46) and neonatal length of stay (*r* = -0.45) two weeks postpartum.
- BSES-SF correlated positively with baby weight at birth (*r* = 0.38).
- ICIQ correlated positively with number of previous deliveries (*r* = 0.33) and number of previous pregnancies (*r* = 0.27).
- MIBS correlated negatively with baby weight (*r* = -0.26).
- EPDS correlated negatively with maternal congenital anomaly details (*r* = -0.22).
- BSES correlated positively with maternal congenital anomaly details (*r* = 0.27).

For the CROM-CROM correlations, the following were observed as shown in Supplementary Table 3:

- A positive correlation was identified between number of previous deliveries and between maternal and neonatal length of stay (*r* = 0.94).
- Negative correlations appeared between route of delivery and postpartum complications (*r* = − 0.49).

## Discussion

This pilot demonstrates preliminary evidence for the feasibility, acceptability, and operational practicality of implementing structured, standardized PROMs and CROMs collection within routine maternity care across diverse private healthcare providers in the Kingdom of Saudi Arabia [9–11]. Achieving an overall response rate of 38.7%, well within international benchmarks of 30–50% for PROMs collection,^12^ indicates that women are willing and able to engage with digital outcome tracking when supported by consistent clinical workflows and accessible platforms [11]. These findings are particularly notable given that this initiative represents one of the earliest large-scale implementations of ICHOM-aligned maternity outcome measurement in the Gulf region.

### Response rate

Response rate variability across hospitals, PROMs types, and CP reflects both expected and context-specific patterns. Higher engagement with psychosocial and social instruments such as EPDS, PHQ-2, SIMSS and SexFS is consistent with global literature showing that questionnaires addressing emotional wellbeing and interpersonal functioning tend to feel more personally relevant and less burdensome. Additionally, patients felt more comfortable filling out these pieces of information without having to directly converse with their physician, avoiding any embarrassment or “taboo” practices [13]. Conversely, lower rates for postpartum instruments—especially at six months—mirror international evidence that maternal engagement decreases as the postpartum period progresses, partly due to competing demands and shifting priorities toward infant care. This underscores the importance of timing, multimodal reminders, simplified interfaces, and embedding PROMs completion into routine maternal visits to reduce attrition.

Additional drivers of response rate performance were also seen throughout the pilot. Provider sites with high leadership commitment, where the OBGYN department chairs, quality teams (including chiefs of quality), and C-suite executives (especially chief medical officers) were actively involved, saw higher PROMs completion rates. In these sites, leadership had continuous follow-up with the implementation teams, reinforced expectations and benefits of the project, and ensured the integration of PROMs into routine clinical practice remained a strategic priority rather than an optional task.

Importantly, sites where physicians routinely reviewed and discussed PROMs results with patients were more likely to maintain sustained response rates. When clinicians clearly saw the value of the data—and explained to patients how their responses informed care planning—PROMs completion shifted from a compliance task to a meaningful feedback loop.

Finally, hospitals that developed clear processes for onboarding and follow-up (e.g., standardized call-back schedules, nurse follow-up scripts) were more successful in securing assessments submitted from home, where drop-off is typically highest.

### MID analysis

The MID analysis provides important insight into the clinical significance of changes observed across the maternity journey. Although mean-level changes across all PROMs remained below MID thresholds—indicating overall stability at the population level—approximately one-third of participating women experienced clinically meaningful improvement in at least one outcome domain. This pattern is consistent with international perinatal research demonstrating heterogeneous postpartum recovery trajectories, where subsets of women achieve substantial physical, emotional, or functional gains even when population averages remain unchanged. The presence of meaningful individual-level improvement despite muted group-level changes reinforces the value of longitudinal PROMs collection for identifying women who benefit most from care, as well as those who may require additional support.

Importantly, these individual-level gains were most evident in hospitals that actively translated PROMs insights into timely, structured clinical responses. PROMs were embedded into routine care through predefined threshold scores within the digital platform, acting as red flags that triggered standardized referral pathways, including pelvic floor physiotherapy for incontinence, mental health escalation for elevated depression scores, and targeted lactation support for low breastfeeding self-efficacy [14,15]. Hospitals that consistently operationalized these pathways demonstrated the greatest improvements across outcome domains.

Providers who introduced pelvic floor rehabilitation referral pathways for patients with abnormal responses saw reductions of up to 70% in urinary incontinence after delivery, while those who did not experienced increases in incontinence rates of up to 90%. Providers who implemented psychiatric referral pathways for patients with abnormal PHQ-2 screening observed reductions of over 60% in depression and anxiety, whereas those without such pathways saw increases in abnormal rates of up to 300% at a single provider site. Similarly, providers who implemented lactation specialist referral pathways demonstrated clear postpartum improvements in breastfeeding self-efficacy, with reductions of up to 70% in abnormal scores, while providers without referral pathways experienced persistent or worsening abnormal BSES rates postpartum, increasing by up to 60%.

By embedding PROMs-driven response pathways in this way, the outcomes program evolved from a measurement exercise into an evidence-based pathway governance model, reinforcing the role of PROMs as decision-enabling tools for personalized maternity care rather than retrospective quality indicators.

### Correlations

Across the maternity PROMs collected, several meaningful correlations emerged. Before birth, measures of overall quality of life (EQ-5D-3L), depressive symptoms (PHQ-2 and EPDS), and sexual functioning (SexFS) showed correlated changes, highlighting how well-being, mood, and interpersonal functioning intersect during pregnancy. After birth, higher breastfeeding confidence aligned closely with stronger bonding and improved postpartum emotional functioning as seen in other studies [16,17].

Correlations between antenatal and intrapartum measures were also observed, where birth satisfaction was strongly linked to whether the mother reported good overall health during pregnancy. Finally, relationships spanning both the pre- and post-birth periods suggest that some postpartum outcomes, such as postpartum depression, may be partially predictable from antenatal PROMs such as SexFS. This indicates that key aspects of the maternal trajectory can, in principle, be identified and acted upon earlier in the care process.

PROM–CROM associations also demonstrated coherent clinical linkages, with higher bowel function during pregnancy correlating with shorter maternal and neonatal length of stay, and breastfeeding confidence correlating with neonatal weight - reinforcing the role of early ambulation after delivery and early baby feeding support in recovery. Parity showed positive associations with both maternal and neonatal length of stay, indicating that having an obstetric history translates to more complex postpartum journeys. The ICIQ showed expected correlations with the number of previous deliveries, indicating increased urinary symptom burden with parity-related pelvic floor strain, consistent with international urogynecology evidence [18]. Although less prominent, congenital anomaly findings displayed negative links with postnatal emotional functioning and breastfeeding confidence measures, underscoring how unexpected neonatal complications shape maternal psychological recovery even when group means remain stable.

The CROM–CROM correlations revealed additional internal consistency across clinical variables, with expected clusters emerging around parity, previous pregnancies, route of delivery, and maternal and neonatal length of stay. Route of delivery, for instance, aligned with patterns of postpartum complication burden—where surgical birth trajectories were linked to increased length of stay—while higher parity was associated with longer maternal and neonatal length of stay, indicating that women with more previous deliveries tended to follow more complex or closely monitored inpatient trajectories as seen in previous studies [19]. These patterns reflect underlying obstetric complexity, the differing profile of patients served at each hospital (including variation in parity, comorbidity burden, and delivery risk), and differences in organizational workflows and processes.

Together, the combined PROM–PROM, PROM–CROM, and CROM–CROM analyses provide a robust multidimensional validation of the data captured in this pilot and illustrate the interconnected nature of clinical outcomes, patient experience, and system-level characteristics.

It is worth noting that the above analysis is based on a limited number of data points at this stage, and stronger correlations may emerge as the dataset grows. Only associations with clear clinical relevance or alignment with established literature were highlighted.

### Limitations

While this pilot demonstrates significant strengths—including multicenter implementation, high PROMs diversity, and rigorous adherence to ICHOM standards—it also has limitations. Response rate variability may introduce selection bias, particularly at later postpartum stages. Clinical data completeness varied by hospital, which could influence specific CROM analyses. Also, one hospital amongst the cohort did not complete the entire pilot and stopped more than midway during the collection phase, which also reduced the overall response rate. Additionally, some MID thresholds were based on our cohort SDs and not previously established scores (due to lack of availability), which may underestimate or overestimate clinical relevance in certain contexts. Nonetheless, these limitations are common in early-stage outcome measurement pilots and provide important learnings for future scale-up.

## Conclusion

The Maternity Outcomes Rollout Pilot is a significant milestone on Saudi Arabia’s Value-Based Healthcare (VBHC) path to 2030. Through the implementation of PROMs and CROMs within routine maternity care, the pilot demonstrated that outcome-based models are both practical and effective in the Kingdom. Providers achieved high participation and uncovered issues of vulnerability that tend to be overlooked in typical care settings, enabling earlier identification of risk-prone women and more targeted, earlier interventions.

Aside from clinical and patient advantages, the pilot built trust between physicians and payers, reframing the relationship around shared objectives of better outcomes, informed choice, and efficiency. The pilot identified the necessity for investment in data infrastructure, standardization of outcomes, and clinician engagement to solidify VBHC adoption and transition towards outcomes-driven care practices.

Operationally, the pilot demonstrated that high-quality outcome collection is achievable when supported by interoperable and unified digital infrastructure, strong clinical leadership and engagement, and culturally attuned patient engagement. From a system standpoint, alignment with national VBHC priorities, emerging accreditation standards, and payment reform initiatives positions PROMs and CROMs integration as a scalable lever to accelerate maternity care transformation across the Kingdom.

Next steps include using the collected data to design value-based payment models for maternity care and expanding this approach to other clinical areas such as diabetes and cardiovascular disease. Integrating PROMs within national frameworks like CHI’s NPROMs strategy will further institutionalize outcome measurement and enable large-scale benchmarking.

This pilot is a proof of concept that the application of VBHC principles and standardized outcome measurement in Saudi Arabia is not just feasible but also clinically significant, enabling scalable evidence-based clinical pathways that deliver sustainable and patient-centered healthcare aligned with Vision 2030.

## Supplementary Information

Below is the link to the electronic supplementary material.

Supplementary Material 1

## Data Availability

No datasets were generated or analysed during the current study.
