# Supplementary material for: A successful model for KSA-based payor-provider collaborative in maternity care
Source: J Patient Rep Outcomes. 2026 Jul 3;10:174. doi: 10.1186/s41687-026-01142-w (PMC13624220; doi:10.1186/s41687-026-01142-w)
Supplement: Supplementary file 1 [file 41687_2026_1142_MOESM1_ESM.docx]

**Correlation Results**

**Table 1. PROM–PROM Correlations**

| **Instrument A** | **Domain A** | **Instrument B** | **Domain B** | **r** | **Direction** |
| --- | --- | --- | --- | --- | --- |
| **EPDS** | Postpartum depression | **PHQ-2** | Antenatal depression/anxiety | **0.59** | ↑ Positive |
| **EPDS** | Postpartum depression | **SexFS** | Sexual function & satisfaction | **0.59** | ↑ Positive |
| **EPDS** | Postpartum depression | **EQ-5D-3L** | General health-related QoL | **−0.57** | ↓ Negative |
| **EQ-5D-3L** | General health-related QoL | **PHQ-2** | Antenatal depression/anxiety | **−0.55** | ↓ Negative |
| **EQ-5D-3L** | General health-related QoL | **SexFS** | Sexual function & satisfaction | **0.48** | ↑ Positive |
| **BSS-R** | Birth satisfaction | **EQ-5D-3L** | General health-related QoL | **0.53** | ↑ Positive |
| **BSS-R** | Birth satisfaction | **EPDS** | Postpartum depression | **−0.55** | ↓ Negative |
| **BSS-R** | Birth satisfaction | **PHQ-2** | Antenatal depression/anxiety | **−0.54** | ↓ Negative |
| **BSES** | Breastfeeding self-efficacy | **EPDS** | Postpartum depression | **−0.38** | ↓ Negative |
| **BSES** | Breastfeeding self-efficacy | **PHQ-2** | Antenatal depression/anxiety | **−0.35** | ↓ Negative |
| **MIBS** | Mother-infant bonding | **BSES** | Breastfeeding self-efficacy | **−0.43** | ↓ Negative |
| **MIBS** | Mother-infant bonding | **BSS-R** | Birth satisfaction | **−0.45** | ↓ Negative |
| **MIBS** | Mother-infant bonding | **EPDS** | Postpartum depression | **0.40** | ↑ Positive |
| **SexFS** | Sexual function & satisfaction | **PHQ-2** | Antenatal depression/anxiety | **0.44** | ↑ Positive |

*Instrument abbreviations: EPDS = Edinburgh Postnatal Depression Scale; PHQ-2 = Patient Health Questionnaire-2; SexFS = PROMIS Sexual Function and Satisfaction; EQ-5D-3L = EuroQol 5-Dimension 3-Level; BSS-R = Birth Satisfaction Scale – Revised; BSES = Breastfeeding Self-Efficacy Scale; MIBS = Mother-Infant Bonding Scale. All correlations reported are statistically significant and clinically relevant. Positive r values indicate that higher scores on Instrument A are associated with higher scores on Instrument B; negative r values indicate an inverse relationship. Correlation method: Spearman's Rank Correlation.*

**Table 2. PROM–CROM Correlations**

| **PROM** | **PROM Domain** | **CROM Variable** | **r** | **Direction** |
| --- | --- | --- | --- | --- |
| **Wexner** | Stool incontinence | Maternal length of stay (2 weeks postpartum) | **−0.46** | ↓ Negative |
| **Wexner** | Stool incontinence | Neonatal length of stay (2 weeks postpartum) | **−0.45** | ↓ Negative |
| **BSES** | Breastfeeding self-efficacy | Baby weight at birth | **0.38** | ↑ Positive |
| **ICIQ** | Urinary incontinence | Number of previous deliveries | **0.33** | ↑ Positive |
| **ICIQ** | Urinary incontinence | Number of previous pregnancies | **0.27** | ↑ Positive |
| **MIBS** | Mother-infant bonding | Baby weight at birth | **−0.26** | ↓ Negative |
| **EPDS** | Postpartum depression | Maternal congenital anomaly (presence) | **−0.22** | ↓ Negative |
| **BSES‡** | Breastfeeding self-efficacy | Maternal congenital anomaly (presence) | **0.27** | ↑ Positive |

*Instrument abbreviations: Wexner = Wexner Stool Incontinence Score; BSES = Breastfeeding Self-Efficacy Scale; ICIQ = International Consultation on Incontinence Questionnaire; MIBS = Mother-Infant Bonding Scale; EPDS = Edinburgh Postnatal Depression Scale. CROM variables are as recorded in the ICHOM Pregnancy and Childbirth Standard Set. Correlation methods varied by variable type: Spearman's Rank Correlation (continuous/ordinal), Point-Biserial Correlation (binary vs. continuous), Phi Coefficient (binary vs. binary).*

**Table 3. CROM–CROM Correlations**

| **CROM Variable A** | **CROM Variable B** | **r** | **Direction** | **Clinical Interpretation** |
| --- | --- | --- | --- | --- |
| **Number of previous deliveries** | **Maternal length of stay** | **0.94** | ↑ Positive | Higher parity linked to longer maternal inpatient stay |
| **Number of previous deliveries** | **Neonatal length of stay** | **0.94** | ↑ Positive | Higher parity linked to longer neonatal inpatient stay |
| **Route of delivery** | **Postpartum complications** | **−0.49** | ↓ Negative | Surgical delivery trajectories associated with increased complication burden |

*Route of delivery is coded as a categorical variable (vaginal vs. caesarean section); correlation computed using an appropriate binary/categorical method (Phi Coefficient or Cramér's V as applicable). All CROM variables are drawn from the ICHOM Pregnancy and Childbirth Standard Set.*
